# Transcriptomic analysis reveals hub genes and subnetworks related to ROS metabolism in *Hylocereus undatus* through novel superoxide scavenger trypsin treatment during storage

**DOI:** 10.1186/s12864-020-06850-1

**Published:** 2020-06-26

**Authors:** Xin Li, Xueru Liu, Xinyue Pang, Yong Yin, Huichun Yu, Yunxia Yuan, Bairu Li

**Affiliations:** 1 College of Food and Bioengineering, Henan University of Science and Technology, No. 263, Kaiyuan Avenue, Luolong District, Luoyang city, 471023 Henan China; 2 State Key Laboratory of Cotton Biology, Henan University, Kaifeng, 455000 China; 3 Key Laboratory of Desert and Desertification, Chinese Academy of Sciences, Lanzhou, 730000 Gansu China; 4 Ministry of Education Key Laboratory of Cell Activities and Stress Adaptations, Lanzhou University, Lanzhou, 730000 China; 5 College of Medical Technology and Engineering, Henan University of Science and Technology, Luoyang, 471023 China

**Keywords:** GO, KEGG, *Hylocereus undatus* (*H. undatus*), Reactive oxygen species (ROS), Protein-protein interaction (PPI), Storage, Trypsin

## Abstract

**Background:**

It was demonstrated in our previous research that trypsin scavenges superoxide anions. In this study, the mechanisms of storage quality improvement by trypsin were evaluated in *H. undatus*.

**Results:**

Trypsin significantly delayed the weight loss and decreased the levels of ROS and membrane lipid peroxidation. Transcriptome profiles of *H. undatus* treated with trypsin revealed the pathways and regulatory mechanisms of ROS genes that were up- or downregulated following trypsin treatment by gene ontology (GO) and Kyoto Encyclopedia of Genes and Genomes pathway (KEGG) enrichment analyses. The current results showed that through the regulation of the expression of hub redox enzymes, especially thioredoxin-related proteins, trypsin can maintain low levels of endogenous active oxygen species, reduce malondialdehyde content and delay fruit aging. In addition, the results of protein-protein interaction networks suggested that the downregulated NAD(P) H and lignin pathways might be the key regulatory mechanisms governed by trypsin.

**Conclusions:**

Trypsin significantly prolonged the storage life of *H. undatus* through regulatory on the endogenous ROS metabolism. As a new biopreservative, trypsin is highly efficient, safe and economical. Therefore, trypsin possesses technical feasibility for the quality control of fruit storage.

**Graphical abstract:**

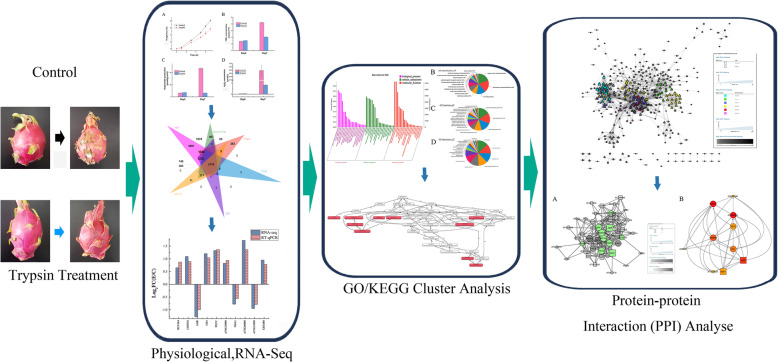

## Background

*Hylocereus undatus* (*H. undatus*) is a perennial climbing cactus plant that is native to tropical areas of Mexico and Central and South America [1]. *H. undatus* is a nonclimacteric fruit that reaches its best edible quality when harvested ripe, and its quality decreases during storage. The shelf life of fresh *H. undatus* is short. As a newly cultivated crop, few studies have aimed to extend the postharvest quality of this fruit [2].

Disorder of reactive oxygen species (ROS) metabolism and excessive accumulation of ROS causes an increase of membrane lipid peroxidation and leads to fruit spoilage during the process of fruit ripening and decay [3, 4]. Trypsin is a serine protease and is used as a proteolytic enzyme. It was shown that the presence of trypsin significantly affects the activity of flavonoids in scavenging 2,2-diphenyl-1- picrylhydrazyl (DPPH), 2, 2-azinobis (3-ethylbenzo- thiazoline-6-sulphonic acid; ABTS) and hydroxyl radicals [5]. We also reported that trypsin can protect cells by scavenging superoxide anions (O_2_^−^) [6]. The function and mechanisms of the impact of trypsin on the quality of *H. undatus* during storage have not been determined to date.

RNA-seq is a highly efficient technology for gene expression analysis [7, 8]. For plants, RNA-seq has been widely used to identify regulatory mechanisms and screen target genes in the area of phytopathology [9, 10]. However, few such studies have been performed with respect to gene expression related to postharvest technology of fruits and vegetables. Although the hub genes involved in the biosynthesis of betalain have been identified in *H. undatus* [11], the regulatory mechanisms of postharvest quality of fruits and vegetables have not been elucidated to date.

The analysis of protein-protein interactions (PPI) enables us to further elucidate the biological processes, organization, and action mechanisms of proteins [12]. Cytoscape is an open platform with a series of plugins to make it more visualized and able to perform deep network analysis. A number of the plugins of Cytoscape, such as NetworkAnalyzer, MCODE, or cytoHubba, could be employed to score and rank the nodes or screen modules in the PPI network [13].

In the current study, we investigated the impact of trypsin on the quality and shelf life of *H. undatus* by regulating active oxygen metabolism. The differentially expressed ROS genes (DERGs) of *H. undatus* peel samples were obtained. GO and KEGG enrichment analyses of DERGs were applied, and the PPI network of ROS related genes and subnetwork of DERGs were constructed. The hub genes related to ROS mechanisms of fruit quality improvement by trypsin during storage were further analyzed by Cytoscape with such plugins as cytoHubba and MCODE.

## Results

### Effect on storage quality of *H. undatus*

**Fig. 1:**
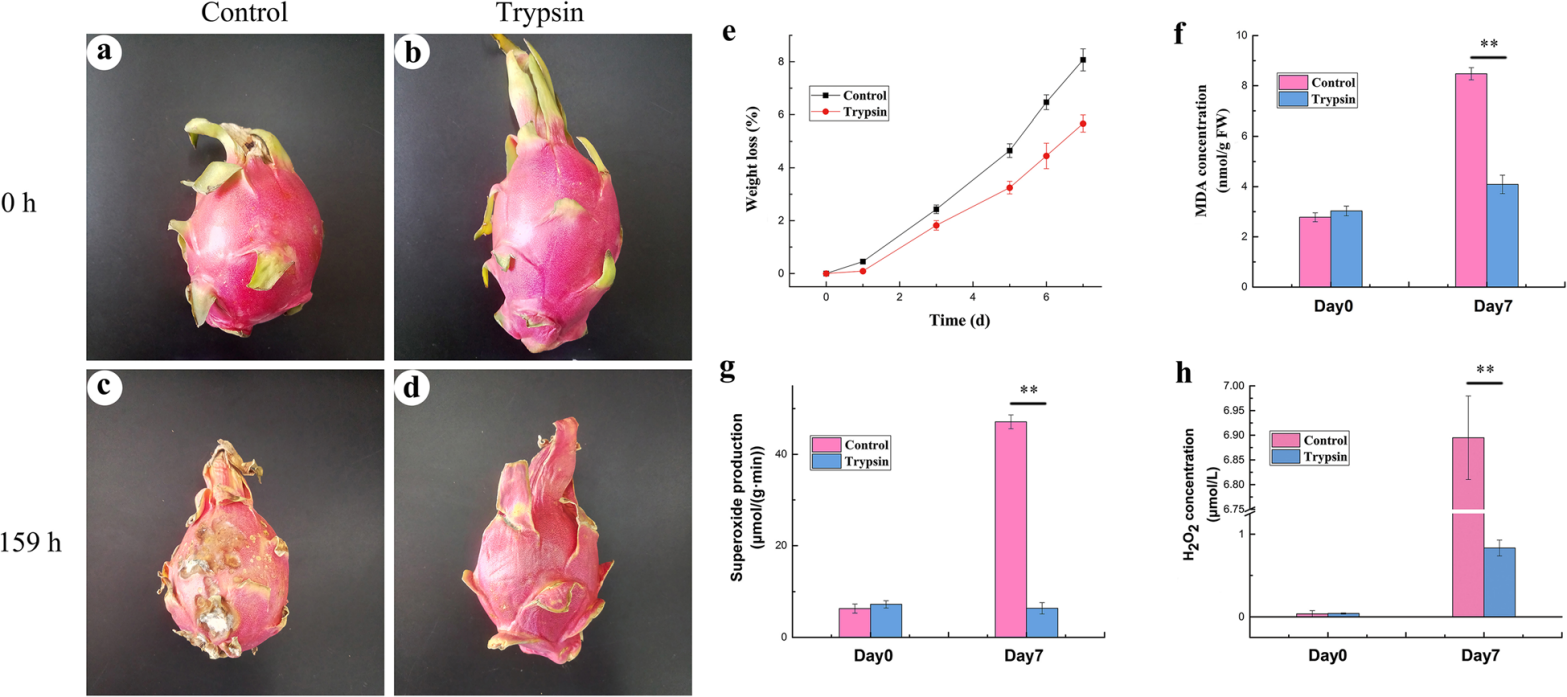
Effect of trypsin treatment on the storage quality or ROS levels of *H. undatus*. Values are the mean ± SE of triplicate samples. Symbols “**” represent *p* < 0.01. **a**-**d**, Fruits of *H. undatus* stored at 25 °C for 159 h with and without trypsin treatment. **a** and **c**, Control fruits; **b** and **d**, trypsin-treated fruits; 0 h or 159 h represent that fruits were stored for 0 or 159 h, respectively. **e**, Weight loss of *H. undatus* fruit stored with or without trypsin for 9 days; **f**, MDA contents of *H. undatus* fruit stored with or without trypsin for 7 days; **g** or **h**, Superoxide anion production rates, or H_2_O_2_ concentrations in *H. undatus* peel with or without trypsin for 7 days

The *H. undatus* fruits of both groups were in excellent condition at the beginning of storage (Fig. 1). After 159 h of storage, the squamas of the fruits in the control group were completely dry and brittle, the color was dim and the fruit bodies were significantly corrupted with plaque and were inedible (Fig. 1c). In the trypsin group, the squamas were partly dry; however, the peel was bright and clean, and the flesh was edible (Fig. 1d).

The weight loss rate of each group showed a significant upward trend with increasing storage time (Fig. 1e). The fruit in the control group exhibited a relatively large mean decrease in fresh weight of 1.15% weight loss per day, while the trypsin group showed only a 0.81% (trypsin) weight loss per day (Fig. 1e). There was a significant difference between the trypsin and control groups (*p* < 0.01).

### Impact on the level of cell injury

To further investigate the preservation mechanism of trypsin, the difference of injury on cells between control and trypsin group was evaluated in *H. undatus*. Figure 1f showed that the membrane lipid peroxidation in the control group sharply increased by fourfold after 7 days of storage. The increase of malondialdehyde (MDA) was fully impeded by trypsin. There was a highly significant difference between the two groups by the end of the storage period (Day 7) (*p* < 0.01).

### Impact on the ROS metabolisms of *H. undatus*

Excess ROS was the major source of cell injury during storage of fruit. Results showed that the levels of O_2_^−^ and H_2_O_2_ in the fruits of the control group increased with storage (Fig. 1 g and h), exhibiting similar trends as did the MDA content. Trypsin entirely inhibited the accumulation of ROS, especially O_2_^−^ (Fig. 1g and h).

### Transcriptomic analysis

#### Sequencing and de novo assembly of Transcriptome

To further reveal the hub genes and key pathway of ROS regulation by trypsin, transcriptomic data was analyzed in *H. undatus*. The two libraries of the control and trypsin groups produced 50,236,685 and 44,897,144 raw reads, respectively (Table 1). The length of a single read was 150 bp. From control and trypsin group libraries, 48,702,393 and 43,456,887 high quality reads were obtained, respectively. Q20 values were 98.31 and 98.33%, respectively. Q30 values were 94.80 and 94.90%, respectively. Also, 224,395 transcripts were composed by high-quality reads using Trinity software. The average length was 1086 bp. N50 value was 1982. The length of transcripts ranged from 201 to 15,462 bp. BUSCO score was 64.8%. Figure S1 showed the length distribution of these transcripts. In fact, 78.63 and 72.92% of transcripts have been mapped, respectively.

**Table 1:** Summary of the sequencing data of *H. undatus*

Sample	Raw reads	Raw bases	Clean reads	Clean bases	Error rate (%)	Q20 (%)	Q30 (%)	GC content (%)	Mapped ratio (%)
Control	50,236,685	7,535,502,700	48,702,393	6,604,345,574	0.013	98.31	94.80	46.87	78.63
Trypsin	44,897,144	6,734,571,600	43,456,887	5,841,023,572	0.012	98.33	94.90	45.19	72.92

#### Functional annotation and analyses

Since there was still no reference genome for *H. undatus*, a total of 131,559 transcripts and 86,808 unigenes were blasted against six public databases (Swiss-Prot, NR, COG, Pfam, GO and KEGG) (E value <1e-4). 67,506 (51.31% of all) transcripts and 31,756 (36.58% of all) unigenes were annotated using these databases (Fig. S2, Table S1 and S2). Based on COG and NOG classifications, 3191 or 2755 unigenes and 8288 or 6677 transcripts were assigned into 24 functional groups, respectively (Fig. S3 and Table S3). The number of transcripts was much higher than that of unigenes. Each unigene was spliced by one or more transcripts. All of these unigenes belonging to three different categories, including biological process (BP), molecular function (MF), or cellular component (CC), have been classified (Fig. S4a).

The main functions were gathered in “binding” and “catalytic activity” on level 2 of molecular function classification (Fig. S4b). In the category of biological processes, they were focused on “organic substance metabolic process” (7758 unigenes, 15.98%), “primary metabolic process” (5442 unigenes, 15.12%) and “cellular metabolic process” (5247 unigenes, 14.54%) (Fig. S4c). In the cellular component, cell part (13.01%) and membrane part (12.35%) were the major parts (Fig. S4d). 7943 unigenes were assigned to 20 s categories belonging to 6 first categories of KEGG pathways (E-value: 1e-4; Identity: 0; Similarity: 0) (Fig. S5 and Table S4).

#### Analysis of differentially expressed genes with trypsin treatment

**Fig. 2:**
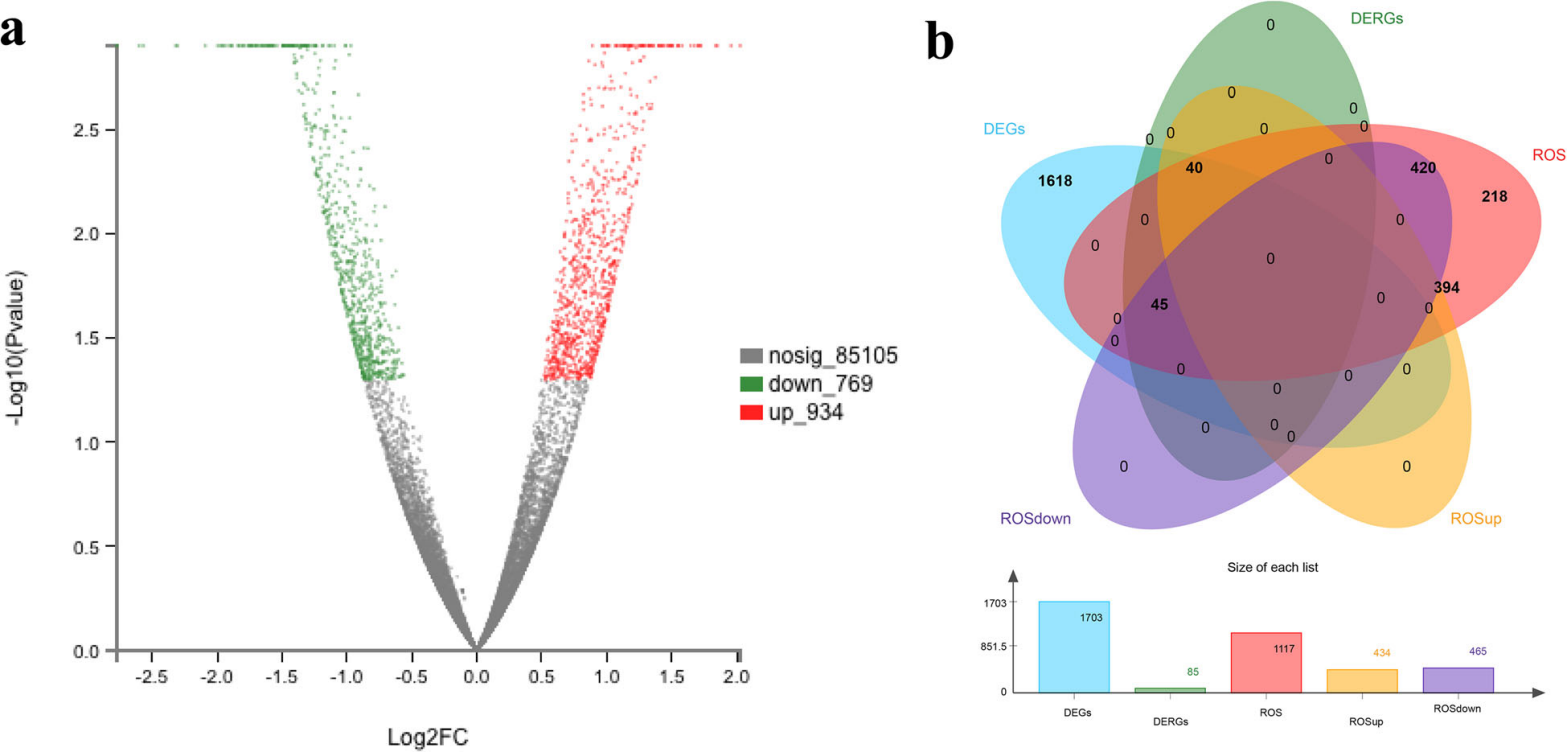
Analysis of differentially expressed genes with trypsin. **a** Volcano plot of significant differences in gene expression between control and trypsin groups; **b** Venn diagram representation of all 1703 differentially expressed genes (DEGs), upregulated genes, downregulated genes, and all of the 1117 ROS related genes (ROSup, ROSdown, and ROS), and 85 DERGs identified in the trypsin treatment group

A total of 31,756 unigenes were identified and quantified from 86,808 genes (Table S1). The expression levels of these genes have been concluded by using a volcano plot (Fig. 2a and Table S5). The total number of different unigenes identified was 1703, including 934 upregulated unigenes (red points) and 769 downregulated unigenes (green points), (*p* < 0.05, fold change (FC) > 1) in the control and treatment groups (Table S5). On the other hand, a total of 1117 ROS related genes (orange circle) were determined, including 434 upregulated unigenes (yellow circle) and 465 downregulated unigenes (purple circle). Among the 1117 ROS related genes, 85 genes (green circle) involved in 1703 genes (blue circle) were expressed to a significantly different extent (Fig. 2b and Table S6).

#### GO enrichment analyses

The biological functions of the patterns up- or downregulated by trypsin treatment were analyzed by gene ontology (GO)-based enrichment. The top 10 GO terms in the two expression patterns were shown in Table 2 (FDR < 10^− 6^). The upregulated enriched GO terms were “oxidoreductase activity, acting on single donors with incorporation of molecular oxygen (GO: 0016701)” and “oxidoreductase activity (GO:0016491).” On the other hand, besides more oxidation/reduction GO terms, “hydrogen peroxide catabolic process (GO: 0042744),” “peroxidase activity (GO: 0004601),” “oxidoreductase activity, acting on NAD(P) H, oxygen as acceptor (GO: 0050664),” and so on, antioxidant or catabolic GO terms, including “peroxidase activity,” “antioxidant activity” or “catalytic activity” were presented in the downregulated pattern (Table S7).

**Table 2:** Top 10 GO terms related to ROS enriched (FDR < 10^−6^) by trypsin

Pattern	Number	GO ID	Term Type	Description	*p*-value^*^	FDR^a^
Upregulation	8	GO:0016701	MF	oxidoreductase activity, acting on single donors with incorporation of molecular oxygen	2.01E-12	1.11E-08
	25	GO:0016491	MF	oxidoreductase activity	3.79E-11	1.05E-07
Downregulation	6	GO:0042744	BP	hydrogen peroxide catabolic process	7.85E-10	6.07E-07
	6	GO:0042743	BP	hydrogen peroxide metabolic process	8.80E-10	6.07E-07
	11	GO:0004601	MF	peroxidase activity	4.05E-12	1.77E-08
	11	GO:0016684	MF	oxidoreductase activity, acting on peroxide as acceptor	6.40E-12	1.77E-08
	11	GO:0016209	MF	antioxidant activity	2.75E-11	5.07E-08
	5	GO:0050664	MF	oxidoreductase activity, acting on NAD(P) H, oxygen as acceptor	5.88E-11	8.06E-08
	28	GO:0016491	MF	oxidoreductase activity	7.30E-11	8.06E-08
	31	GO:0003824	MF	catalytic activity	1.80E-10	1.65E-07

^*^p-values were calculated using Fisher’s test

^a^FDR corrections were calculated using the Benjamini-Hochberg procedure

**Fig. 3:**
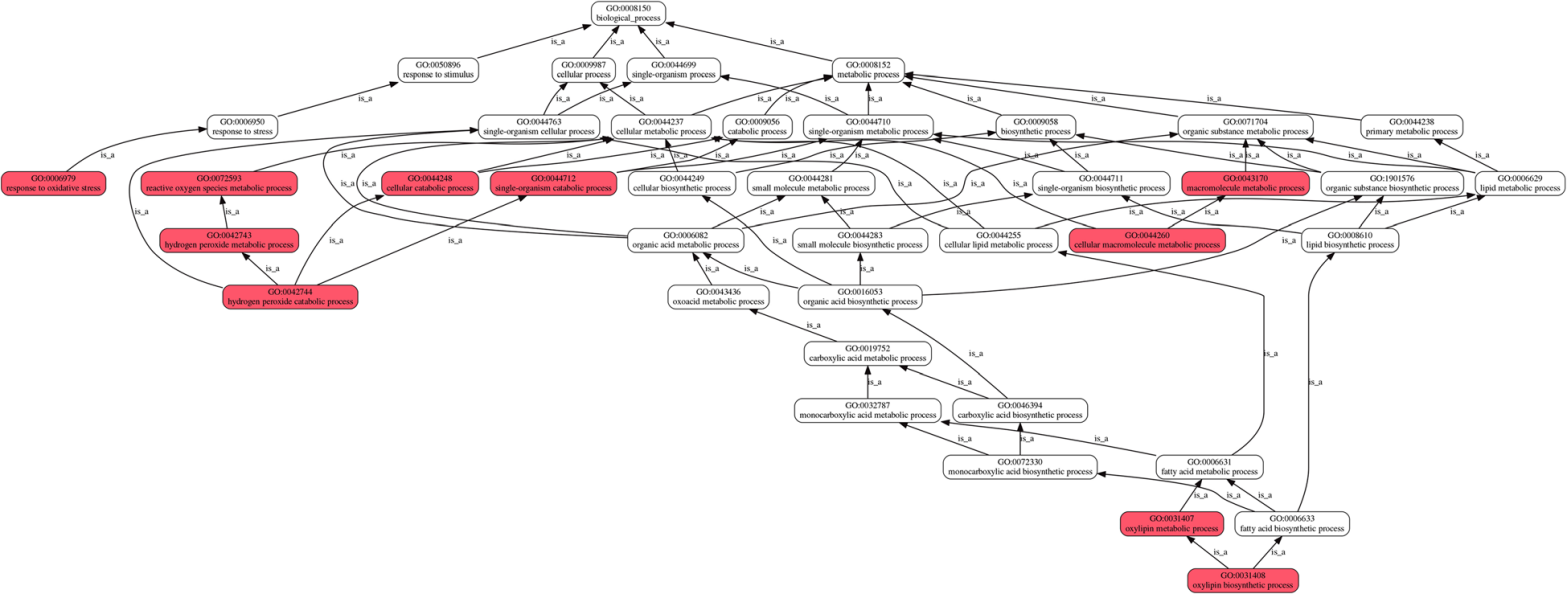
Relationships between GO terms in a Directed Acyclic Graph (DAG). Red to white colors represent decreasing significance levels (Red is the most, while white is the least significant). The figure illustrates a subset of the molecular function DAGs for the oxylipin biosynthetic process (GO:0031408), hydrogen peroxide catabolic process (GO:0042744) and response to oxidative stress (GO:0006979). Arrows indicate relationships of the **is_a** type. The ancestors of GO:0031408, GO:0042744 or GO:0006979 are highlighted back to the root of the biological process ontology via arrows

The major ROS related pathways involved in trypsin regulation can be summarized into a schematic representation (Fig. 3). GO terms are related to one another in a directed acyclic graph (DAG), where more detailed terms are described as children of more general terms. For example, the GO biological process “hydrogen peroxide catabolic process (GO:0042744)” is a child of four terms: “single-organism cellular process (GO:0044763),” “hydrogen peroxide metabolic process (GO:0042743),” “cellular catabolic process (GO:0044248),” and “single-organism catabolic process (GO:0044712).” The GO biological process “oxylipin biosynthetic process (GO:0031408)” is a child of two terms: “oxylipin metabolic process (GO:0031407)” and “fatty acid biosynthetic process (GO:0006633).” These in turn have parent terms, as shown in Figure 3, tracing back to the ultimate ancestor, biological process (GO:0008150), the root of the molecular function ontology. In addition, H_2_O_2_ catabolic metabolism was only downregulated (Fig. S6). Cell redox homeostasis was shown to be an upregulated GO process (Fig. S7).

#### KEGG pathway analyses

The top 9 enriched pathways were shown in Table 3 (FDR < 0.05). With trypsin, downregulated ROS related genes were enriched in several pathways, including the “phenylpropanoid biosynthesis pathway (map 00940),” which is associated with fatty acid biosynthesis, as shown in the GO analysis section, “MAPK signaling pathway - plant (map 04016),” which involves a series of defense responses induced by ROS, and “Plant-pathogen interaction (map 04626),” which highly focus on the hypersensitive response (HR) induced by ROS. On the other hand, “Linoleic acid metabolism (map 00591),” “Photosynthesis (map 00195),” “Ascorbate and aldarate metabolism (map 00053),” and “Porphyrin and chlorophyll metabolism (map 00860)” were significantly upregulated.

**Table 3:** Pathways related to ROS enriched (FDR < 0.05) by trypsin

Pattern	Number	KO ID	Term	*p*-value^*^	FDR^a^
Upregulation	3	map 00591	Linoleic acid metabolism	1.56E-05	0.00034
	3	map 00195	Photosynthesis	0.00017	0.0018
	2	map 00053	Ascorbate and aldarate metabolism	0.0084	0.046
	2	map 00860	Porphyrin and chlorophyll metabolism	0.0065	0.047
Downregulation	9	map 00940	Phenylpropanoid biosynthesis	1.29E-10	2.06E-09
	6	map 04016	MAPK signaling pathway - plant	5.52E-06	4.41E-05
	6	map 04626	Plant-pathogen interaction	1.34E-05	7.15E-05
	2	map 00073	Cutin, suberin and wax biosynthesis	0.0020	0.0080
	2	map 00592	alpha-Linolenic acid metabolism	0.010	0.033

^*^p-values were calculated using Fisher’s test

^a^FDR corrections were calculated using the Benjamini-Hochberg procedure

#### PPI networks of DERGs

In total, we obtained 85 DERGs (FDR < 0.01, 40 upregulated and 45 downregulated) among 1117 ROS related genes, including 434 upregulated genes and 465 downregulated genes (Table S8).

**Fig. 4:**
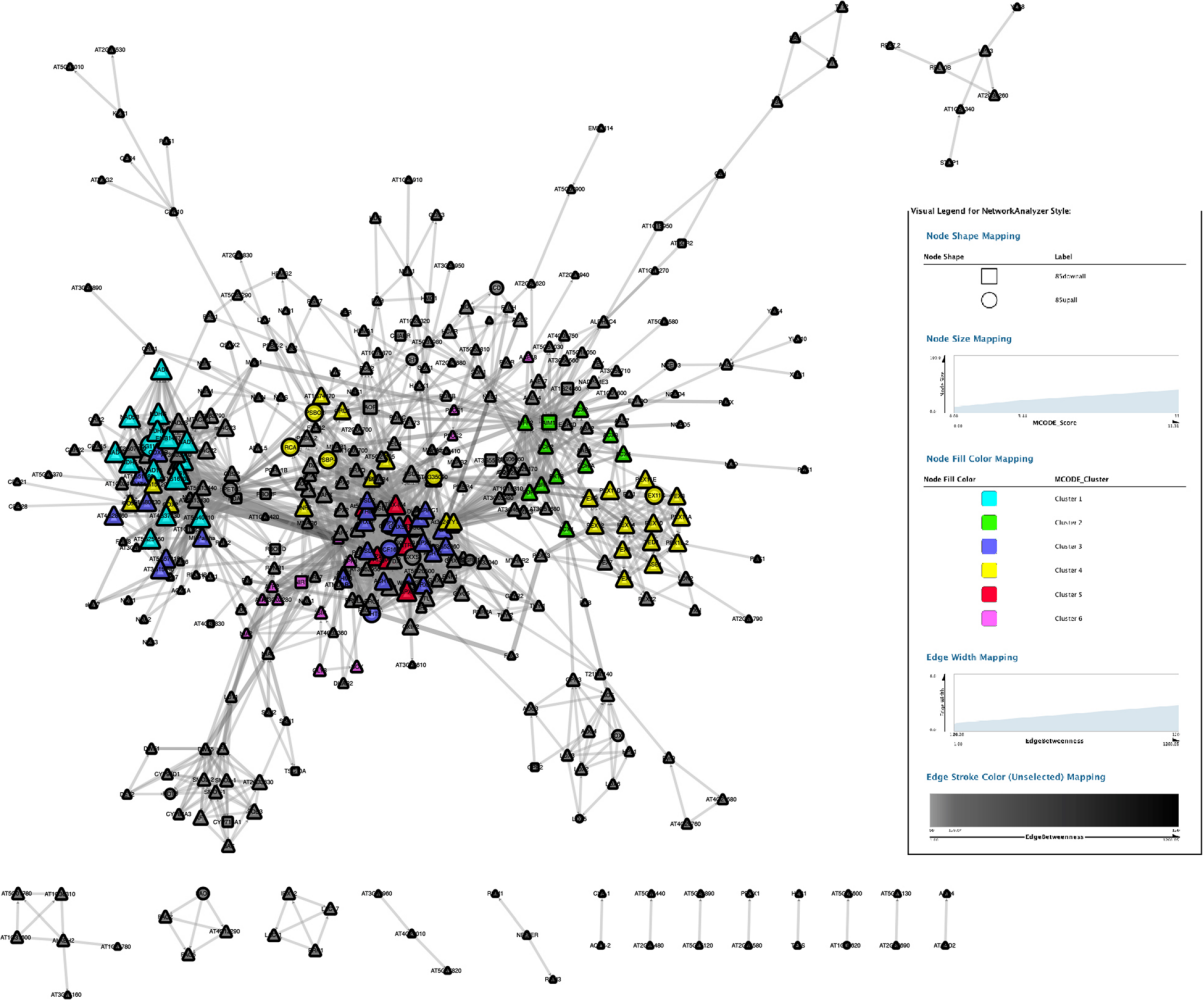
ROS related PPI networks induced by trypsin were constructed by Cytoscape software. Rectangle nodes represent proteins encoded by downregulated genes; Round nodes represent proteins encoded by upregulated genes. Triangle-shaped nodes represent the last interacting proteins without significantly different expression. The top 6 clusters calculated by MCODE were colored as shown in the legend

The PPI subnetwork of total ROS genes was composed of 404 nodes and the first 2000 edges, containing 40 DERGs (Fig. 4 and Table S9). The upregulated ROS gene PPI subnetwork contained 278 nodes and the first 2000 edges, including 29 upregulated DERGs (Fig. S8a and Table S10). The downregulated antioxidant gene PPI subnetwork contained 288 nodes and the first 2000 edges (interactions), including 10 downregulated DERGs (Fig. S8b and Table S11).

**Fig. 5:**
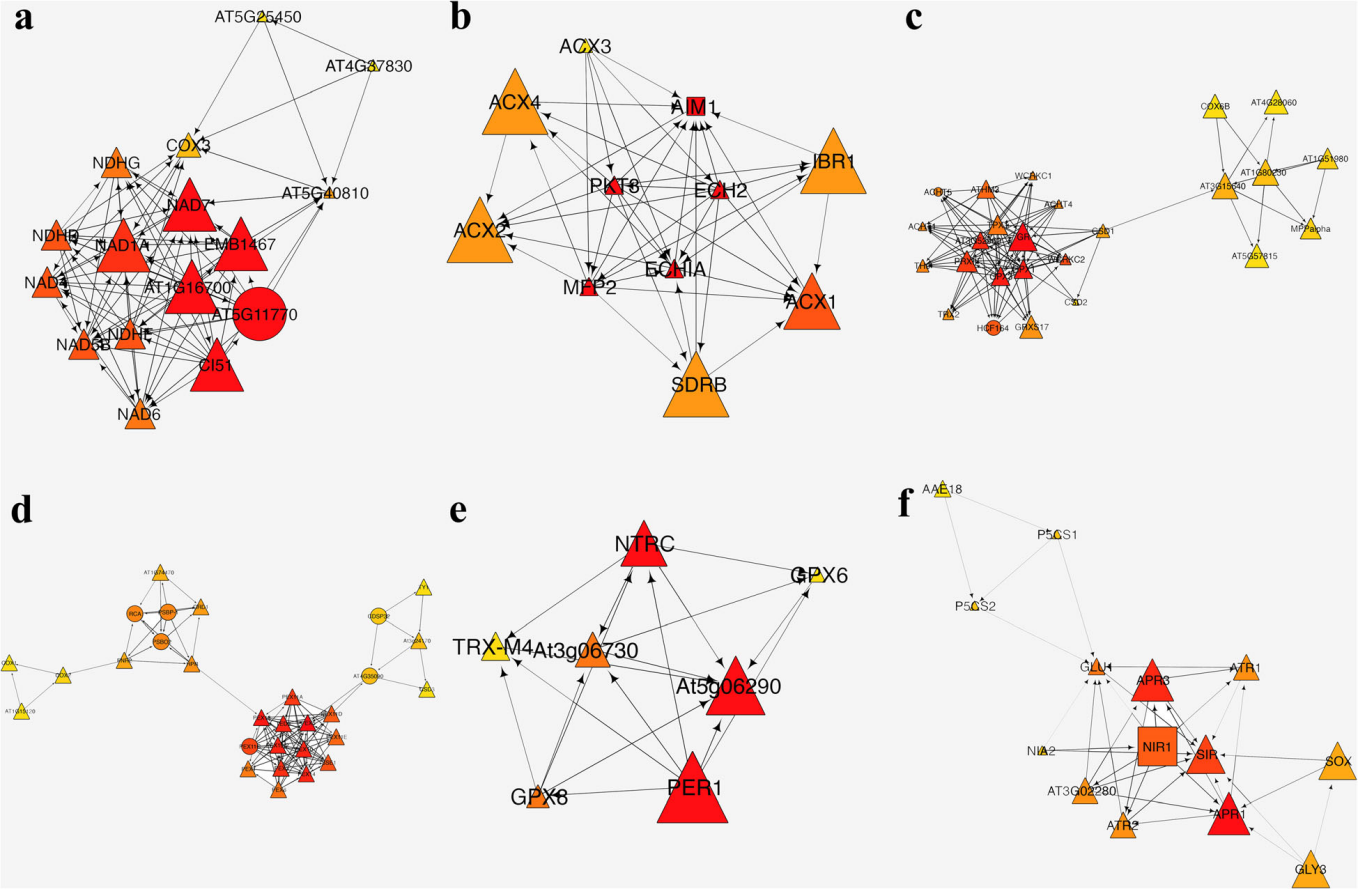
Top 6 clusters of ROS related PPI network calculated by MCODE. **a**-**f** represent clusters 1–6, respectively

Further, the Cytoscape plugin “MCODE” was layered on the PPI network. Twenty clusters were obtained (Table S12). Nodes belonging to the top 6 clusters were labeled by different colors in the PPI network (Fig. 4). The top 6 clusters analyzed by the CytoHubba plugin of Cytoscape were then shown in Figure 5. The central nodes of each cluster were shown in Table 4.

**Table 4:** Central nodes of top 6 clusters of ROS related PPI subnetwork of *H. undatus*

Cluster	Central nodes	Score of clusters	Score of each node
1	AT1G16700; NAD7; EMB1467; AT5G11770; CI51	10.533	322,680
2	PKT3; ECHIA; MFP2; AIM1; ECH2	8.600	2280
3	GR	7.750	344
4	PEX5	7.571	171,362
5	At5g06290; PER1; NTRC	6.000	72
6	APR1	5.385	42

**Fig. 6:**
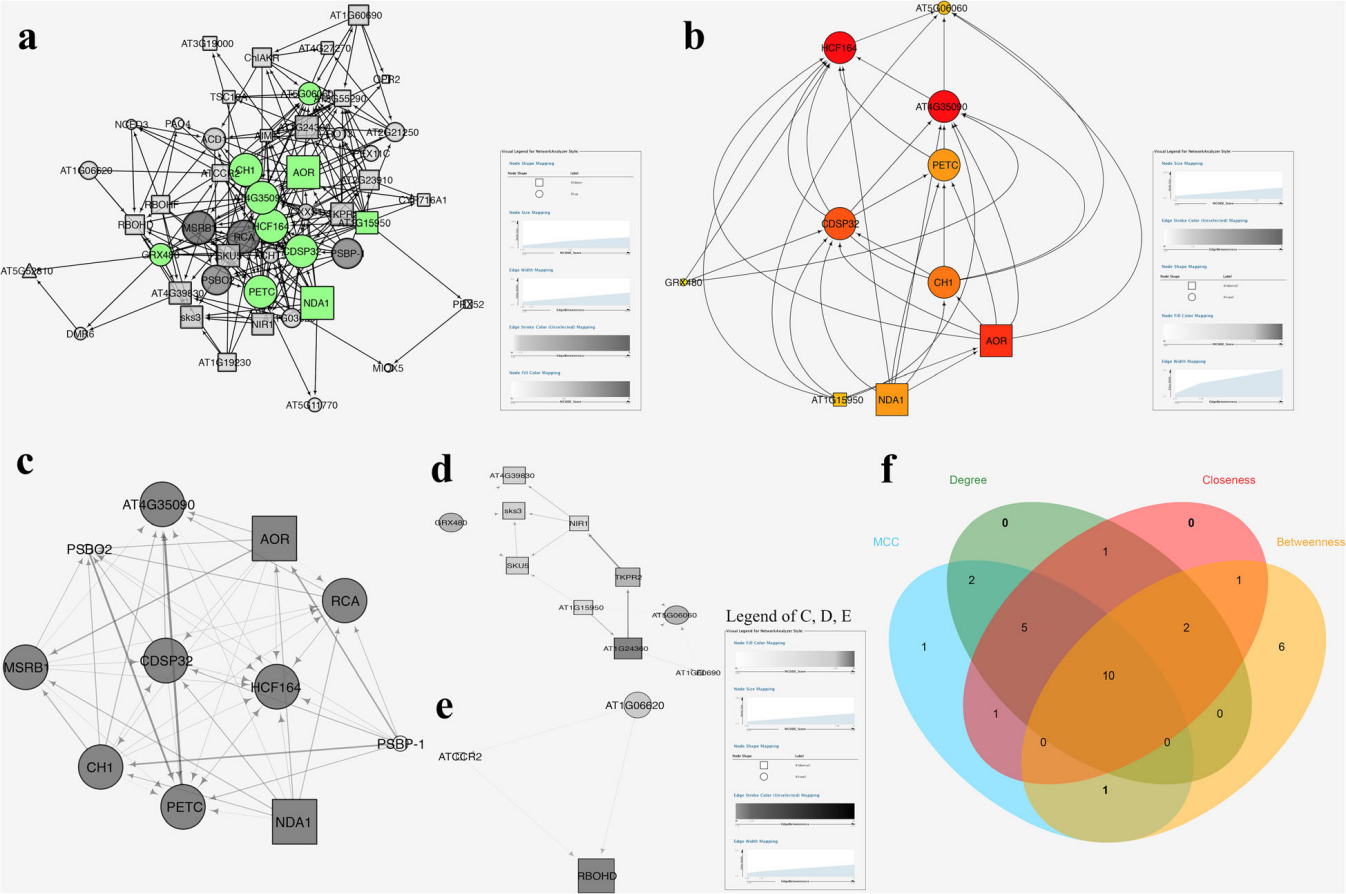
Analysis of hub genes of DERGs. The shape setting of nodes was the same as in Figure 4. **a**, Subnetwork of DERGs. The top 10 nodes of cytoHubba were colored; **b**, Hierarchical layout of 10 hubs; **c**-**e**, Clusters 1–3 of DERGs by MCODE; **f**, Overlapping DERGs among the four methods according to cytoHubba

Furthermore, all of the DERGs were selected to construct 3 new PPI networks, including total (60 nodes, 255 edges) (Fig. 6a), upregulated (30 nodes, 81 edges) (Fig. S9a), and downregulated (28 nodes, 62 edges) DERGs subnetworks (Fig. S9b). Three clusters were constructed by MCODE as shown in Figure 6 c, d and e.

Based on the calculation of CytoHubba plugin, because the screen results of either Density of Maximum Neighborhood Component (DMNC) or Maximum Neighborhood Component (MNC) did not match well to the other methods, hub genes were determined by overlapping the genes according to 4 ranked methods, including Maximal Clique Centrality (MCC), Degree, Closeness and Betweenness in cytoHubba (Table S13). Ten hub genes were discovered, including 7 upregulated and 3 downregulated genes (Fig. 6 b and f).

#### Topological properties of networks

The node degree distributions of the total, upregulated, and downregulated ROS related gene subnetworks followed power law fit distributions (*R*^2^ = 0.798, 0.765, and 0.762, respectively) (Fig. S10). The subnetwork topological parameters, including network centralization clustering coefficient, and so on, were shown in Table 5.

**Table 5:** Topological parameters of the ROS related genes, DERGs and Hub genes of the PPI subnetwork of *H. undatus* impacted by trypsin

PPI subnetwork	y = βx^α^	R^2^	Correlation	Clustering coefficient	Network centralization	Network density	Num. of nodes	Characteristic path length
ROS related genes network								
Total ROS	y = 137.73x^-1.213^	0.798	0.891	0.381	0.150	0.025	404	3.920
Upregulated ROS	y = 52.493x^-0.889^	0.765	0.853	0.413	0.286	0.052	278	2.839
Downregulated ROS	y = 60.805x^-0.931^	0.762	0.733	0.353	0.299	0.048	288	2.748
DERGs of ROS subnetwork								
Total DERGs	y = 7.431x^-0.521^	0.412	0.582	0.361	0.395	0.144	60	2.397
Upregulated DERGs	y = 4.697x^-0.532^	0.422	0.807	0.354	0.355	0.186	30	2.448
Downregulated DERGs	y = 3.289x^-0.026^	0.001	−0.021	0.239	0.222	0.164	28	2.437
Hubs subnetwork								
Total 10 hubs	y = 0.688 × ^0.468^	0.268	0.499	0.849	0.333	0.733	10	1.267

The subnetworks of DERGs, except the downregulated DERGs subnetwork (*R*^2^ = 0.001), were characterized as scale-free networks, even though they were much weaker than ROS related genes networks, which approximately followed power law fit distributions, with *R*^2^ = 0.412 and 0.422, respectively (Fig. S10 and Table 5). Since the DERGs were chosen from the ROS related genes network, either the centralization or the density of the total DERGs network was much higher (0.395 and 0.144, respectively) than that of the total ROS related genes network (0.150 and 0.025, respectively). Finally, the correlation of hub network was decreased to 0.499 (*R*^2^ = 0.268).

#### Accuracy of RNA-Seq data verification by RT-qPCR

The accuracy of the RNA-Seq data of ten hub genes of DERGs involved in ROS metabolism were verified by reverse transcription quantitative PCR (RT-qPCR) (Fig. S11). The IDs, NR description, Log_2_FC(E/C), *p* value, FDR, and primers of the 10 hub genes are shown in Table 6 and Table S16.

**Table 6:** Primer sequences used in RT-qPCR

Name	Gene ID	Primer
β-actin		Forward: 5′-TCTGCTGAGCGAGAAAT-3′
		Reward: 5′-AGCCACCACTAAGAACAAT-3′
HCF164	TRINITY_DN39264_c0_g2	Forward: 5′-GCGAATGATAATCCAAGCCAAGC −3′
		Reward: 5′-TATGATACCGCCAAGGCAGACAG −3′
CDSP32	TRINITY_DN42075_c0_g1	Forward: 5′-TTCGCTTCTCCTTTCTCCCTTGC-3′
		Reward: 5′-TGCTGTGGACTTTCTGGACCCTC-3′
AOR	TRINITY_DN44827_c0_g2	Forward: 5′-GAGTACACGGCTGCTGAAGAAAG-3′
		Reward: 5′-GGCACCACCAAGAATAAGGATAG-3′
CH1	TRINITY_DN40044_c0_g1	Forward: 5′-AGGTTAGAGGCAACATTGGAGTC-3′
		Reward: 5′-ATGGAGCATCATTATGGTGAGAA-3′
PETC	TRINITY_DN41555_c0_g1	Forward: 5′-CCCATCAACAGCGGTGGCTAAAC-3′
		Reward: 5′-GGGACGAAGAAGGCAGCGTAAGG-3′
AT4G35090	TRINITY_DN52071_c2_g1	Forward: 5′-GCAGCCCTGAAACCATCCGTGAC-3′
		Reward: 5′-CCCACATCGTCGAAGAGCCAACC-3′
NDA1	TRINITY_DN41480_c0_g1	Forward: 5′-GAGAGAGAGCCAATATCCTATGAAGCATCC-3′
		Reward: 5′-GGATGCTTCATAGGATATTGGCTCTCTCTC-3′
AT5G06060	TRINITY_DN39500_c0_g1	Forward: 5′-GGGTCTCAGGAAAGAAACAGTAA-3′
		Reward: 5′-GCTCCATAAATAGGACCAGCATTA-3′
AT1G15950	TRINITY_DN6609_c0_g1	Forward: 5′-AAAGAATGCCCATTTGAGGGAGC-3′
		Reward: 5′-TTTGTGCCGTTTACTGCTGGTTC-3′
GRX480	TRINITY_DN13090_c0_g1	Forward: 5′-AACCACCTTACCAAGAGCTTCCC-3′
		Reward: 5′-CCAATAACAAACGCCTGACAACAT-3′

## Discussion

Trypsin treatment alone can already significantly reduce the loss of water, impede the dehydration and improve the fruit quality. MDA is considered to represent the degree of cell membrane lipid peroxidation, since it is a marker of membrane lipid peroxidation [14]. The result of MDA indicates that trypsin can significantly decrease the MDA content, which represents the lipid peroxidation of the cell membrane, thereby significantly slowing the cell damage.

In the process of maturity or decline, the disruption of balance between the generation and scavenging of ROS causes the accumulation of ROS. A high concentration of ROS such as O_2_^−^ and H_2_O_2_ can cause lipid peroxidation, which is the main cause of membrane damage [15]. As expected, the novel superoxide scavenger trypsin entirely inhibited the accumulation of ROS, especially O_2_^−^. However, how can trypsin perform ROS regulation? Which genes were impacted by trypsin during storage? The transcriptomic analysis was used to reveal the hub genes regulating ROS metabolism through trypsin treatment.

Analyzed from transcriptomic profile, a total of 1117 ROS related genes were determined, including 434 upregulated unigenes and 465 downregulated unigenes. Among the 1117 ROS related genes, 85 genes were expressed to a significantly different extent.

The biological functions of the patterns up- or downregulated by trypsin treatment were analyzed by gene ontology (GO)-based enrichment. Lots of oxidation/reduction GO terms were enriched. The major ROS related pathways involved in trypsin regulation were summarized by DAGs (Fig. 3). The DAGs indicated that the H_2_O_2_ catabolic metabolism and oxylipin biosynthesis are key processes of trypsin regulatory mechanisms during *H. undatus* storage.

Next, to illustrate the pathways involved in the trypsin responsive patterns, the KEGG pathways were enriched. Important physiological metabolic processes, such as photosynthesis, porphyrin and chlorophyll metabolism, were induced, while defense responses were impeded by trypsin. In the top 2 pathways regulated by trypsin, 11 and 7 genes were involved in “Phenylpropanoid biosynthesis (map 00940)” and “MAPK signaling pathway- plant (map 04016)”. As we know, peroxidase (POD) is the key enzyme of the last process of lignin synthesis [16, 17]. In map 00940, Guaiacyl lignin, 5-hydroxy-gualacyl lignin, syringyl lignin and p-hydroxy-phenyl lignin were regulated by POD (Fig. S12). In map 04016, results showed that respiratory burst oxidase (RbohD) was regulated to maintenance the homeostacis of ROS, and stress-tolerant response was also induced which due to the significantly activated catalase (CAT) (Fig. S13). These observations were consistent with our previous results [18]. The results of KEGG indicated that, as a novel superoxide scavenger, trypsin regulated antioxidant system of pitaya and exhibited protection of pitaya during storage.

It is essential to explore the potential ROS regulatory mechanisms of trypsin by the exposition of the DERGs; the investigation on the PPI networks would promote the functional research on DERGs induced by trypsin. The PPI network of total, upregulated, or downregulated ROS genes were constructed. These three subnetworks indicated that trypsin treatment greatly disturbed the PPI network in *H. undatus.* The biological consequences were magnified by hundreds of proteins interacting with DERGs.

Further, the Cytoscape plugin “MCODE” and “CytoHubba” were layered on the PPI network to illustrate subnetworks and screen hub genes. Among the obtained 10 hub nodes, 7 nodes, including HCF164 (Thioredoxin-like protein), AT4G35090 (Catalase), PETC, and so on, were clustered in module 1 of the DERGs PPI network; the other 3 nodes including AT5G06060 (NAD(P)-binding Rossmann-fold superfamily protein), GRX480 (Thioredoxin superfamily protein), and AT1G15950 (Cinnamoyl-CoA reductase) were clustered in module 2 of the DERGs PPI network (Table S14 and S15). In addition, only HCF164 was clustered in the top 6 modules (cluster 3) of all ROS related PPI networks (Figs. 5 and 6). Most of the hubs were thioredoxin related proteins. This result indicated that the mechanisms of trypsin regulation of ROS are closely associated with sulfur metabolism.

Furthermore, the hub of AT1G15950 (Cinnamoyl-CoA reductase) suggested that the quality of *H. undatus* storage was associated with lignin metabolism, which was also shown in the DAG of GO analysis (Fig. 3). From the information of the arrow directions, either NDA1 (Internal NAD(P) H dehydrogenase in mitochondria) or AOR (NADPH-dependent alkenal/one oxidoreductase, chloroplastic) is the upstream gene of AT5G06060 (Fig. 6). This pathway is dependent on the redox of NAD(P) H, which is the main source of O_2_^−^. These results strongly indicated that the downregulated NAD(P) H and lignin pathways might be the key regulatory mechanisms of trypsin, the superoxide scavenger, on the quality improvement of *H. undatus* during storage. While, there still lots of question unclear. For example, who is the transcriptional regulation factor primarily induced by trypsin? Further works are needed to investigate the regulatory mechanisms of trypsin on lignin synthesis.

The correlation of the distribution of node degrees of a PPI network to the power law distribution is a judgment standard for scale-free networks. This property distinguishes the PPI network from random networks [19]. The node degree distributions of the total, upregulated, and downregulated ROS related gene subnetworks followed power law fit distributions. This suggested that these three PPI subnetworks were true cellular complex biological scale-free networks. These results also showed that a few protein nodes serve as hubs with links to other protein nodes [19].

As expected, the correlation of hub network was decreased with the decrease of nodes of total ROS, DERGs, and hub networks (Table 5). This indicates that the hub genes are highlighted in a larger network and that our PPI networks are reliable.

The accuracy of the RNA-Seq data of these hub genes were verified by RT-qPCR. Expression changes of these 10 genes were consistent with the RNA-Seq data. This showed that RNA-Seq data are credible.

## Conclusions

Trypsin treatment significantly reduced the accumulation of ROS, including O_2_^−^ and H_2_O_2_, in *H. undatus* during storage, impeded membrane lipid peroxidation, and prolonged the storage life of *H. undatus*. Transcriptomic analysis revealed 10 hub genes regulated by trypsin involved in *H. undatus* quality improvement during storage. PPI network analysis suggested that the downregulated NAD(P) H and lignin pathways might represent the key regulatory mechanisms of trypsin. As a new biopreservative, trypsin is highly efficient, safe and economical. Therefore, trypsin possesses technical feasibility for the quality control of fruit storage.

## Methods

### Main materials

*H. undatus* (Vietnam No. 1 cultivar, white pulp) was harvested from Ruyang county in Henan Province, China. The plant was identified by Prof. Zhaoyong Shi (College of Agriculture, Henan University of Science and Technology, Luoyang, China) and the voucher specimens (No. Hu-20,160,923) have been deposited in our laboratory. Fruits without mechanical damage and with uniform color, size and number of scales were chosen for the study. Trypsin (bovine, 500 units/mg, crystalline) was purchased from Ameresco (Solon, OH, USA).

### *H. undatus* treatment methods

Trypsin was brushed evenly for 80 s onto the peels of 15 *H. undatus* fruits as the trypsin group. The fruits of *H. undatus* of the control group were treated in the same conditions with PBS buffer. The fruits were then placed in an incubator (25 °C, 85% relative humidity), and their physical and chemical indices (weight loss, MDA, etc.) were periodically measured. The optimal concentration of trypsin (2.41 × 10^− 6^ mol/L) was determined in our pre-experiment and used for the current study.

### Library construction and Illumina RNA-sequencing

Total RNA was extracted from two groups of *H. undatus* peels (with or without trypsin). The peels of each group were taken from 15 fruits. Transcriptome libraries for RNA-seq were constructed from 5 μg samples of RNA (≥ 100 ng/μL) using the Truseq™ RNA sample preparation kit from Illumina (San Diego, CA). Libraries were constructed and sequenced with the Illumina HiSeq xten (2 × 150 bp read length) by the Tolo Biotech company of China. All data were uploaded to the I-Sanger cloud platform and analyzed as described below.

### De novo assembly and annotation

The raw paired end reads were trimmed and quality controlled by SeqPrep (https://github.com/jstjohn/SeqPrep) and Sickle (https://github.com/najoshi/sickle) with default parameters. Then clean data from the samples were used to do de novo assembly with Trinity (http://trinityrnaseq.sourceforge.net/) [20]. All the assembled transcripts were searched against the NCBI protein nonredundant (NR), String, and KEGG databases using BLASTX to identify the proteins that had the highest sequence similarity with the given transcripts to retrieve their function annotations and a typical cut-off E-values less than 1.0 × 10^− 5^ was set. BLAST2GO (http://www.blast2go.com/b2ghome) program was used to get GO annotations of unique assembled transcripts for describing biological processes, molecular functions and cellular components. Metabolic pathway analysis was performed using the Kyoto Encyclopedia of Genes and Genomes (KEGG, http://www.genome.jp/kegg/).

### Identification of differentially expressed genes

To identify differentially expressed ROS genes (DERGs) between control and samples treated with trypsin, the expression level of each transcript was calculated according to the fragments per kilobase of exon per million mapped reads (FRKM) method. Differential expression analysis was performed by EdgeR software from the R statistical package. The false discovery rate (FDR) was used to adjust the resulting *P*-values using the Benjamini and Hochberg approach.

### GO and KEGG enrichment for differentially expressed genes

Functional-enrichment analyses including GO and KEGG were performed as reported by Candar-Cakir et al. [21]. The enriched GO terms were shown with DAGs (directed acyclic hierarchical graph) and bar charts [21].

### Gene expression analysis by reverse transcription-qPCR

Total RNA was extracted as described above. Reverse Transcription-qPCR was performed as reported by Yang et al. [22]. The information of primers is listed in Table 6. β-actin was used as internal control. The relative copy numbers of the genes were obtained by the 2^−∆∆Ct^ method [10, 22].

### Protein-protein interaction (PPI) analyses

#### PPI network generation

The ROS related proteins were screened from the I-Sanger cloud platform and imported into Cytoscape software. The relationship between ROS related proteins and their putative targets was visualized through PPI networks of ROS, DERGs, or Hubs of *H. undatus* induced by trypsin using Cytoscape [19].

#### Network topological parameters

Several network topological parameters could compare and characterize the complex networks. The primary topological parameters of networks were calculated by NetworkAnalyzer [19]. Here, the edges were considered as undirected. The equation y = βx^α^ and parameters R^2^ and correlation described the fit to the power line [23].

#### Module

A Molecular Complex Detection (MCODE) analysis was performed to identify the clusters in the entire ROS related network (Degree cutoff: 2; Node score cutoff: 0.2; K-Core: 2; Max depth: 100) [24]. Here, the edges were treated as directed for MCODE or next hub node analysis.

#### Analysis of hubs

The key genes in the PPI network were investigated topologically by NetworkAnalyzer. The cytoHubba plugin of Cytoscape further analyzed the network, and the high degree nodes were identified [25]. Through the cytoHubba plugin, 11 topological analysis methods were obtained. To increase the sensitivity and specificity, besides three centrality methods including degree, closeness, and betweenness centrality, the top 20 hub-forming proteins were also identified based on local method, including MCC, MNC and DMNC, respectively. Then, the overlapping proteins were considered as the hubs. Finally, we found the common nodes using Venn diagrams [26].

### Determination of the weight loss rate

The weight loss rates for each group were determined using 3 replicates (*n* = 6) and were recorded at the same time every day for 9 days as the fruit was stored at 25 °C. The replicate information, including recording time point or temperature, among others, was identical to that of other index determinations, including browning index and electrical conductivity. The percentage of weight loss was calculated after days of storage.

### Quantification of lipid peroxides in *H. undatus* peel

MDA contents were measured using the thiobarbituric acid reactive substrates (TBARS) assay as reported by Zhou et al. (2014) [27].

### Determination of superoxide and hydrogen peroxide content of *H. undatus* peel

First, 2 g of *H. undatus* peel was ground with 6 ml 50 mM PBS (pH = 7.8) and 1% (w/v) PVP at 0 °C. The sample was obtained by 12,000 g centrifugation (4 °C, 15 min). The production of O_2_^−^ and hydrogen peroxide (H_2_O_2_) was as described by Schneider et al. [28] or Li and Imlay in 2018 [29], respectively.

### Statistical analyses

The SPSS statistical software package (11.0.1) (15 November 2001, SPSS Inc., Chicago, IL) was used for data analyses. Our results were obtained by 3 independent experiments. A paired sample t-test was used to analyze the differences between samples. Significant difference was estimated by *p* < 0.05. Highly significant means *p* < 0.01.

## Supplementary information

**Additional file 1: Fig. S1**. Statistics of assembly length of transcripts. **Fig. S2.** Venn diagram of sequence statistics of functional annotation of RNA-Seq data for each database. **Fig. S3.** COG and NOG classification of transcripts or unigenes. **Fig. S4.** GO classification of unigenes. **a** Bar chart of three categories; **(b-d)** Molecular Function (MF), Biological Process (BP), or Cellular Component (CC) categories of unigenes. **Fig. S5.** Histogram of KEGG terms associated with trypsin. Different categories of KEGG terms were shown in different color. Red, Metabolism; Green, Genetic Information Processing; Purple, Environmental Information Processing; Blue, Cellular Processes; Yellow, HD, Organismal Systems; Brown, Human Diseases. **Fig. S6.** Relationships between GO terms of downregulated DERGs in a Directed Acyclic Graph **(DAG).** The information of color and arrow was same to that in Fig. 3. **Fig. S7.** Relationships between GO terms of upregulated DERGs in a Directed Acyclic Graph (DAG). The information of color and arrow was same to that in Fig. 3. **Fig. S8**. PPI network of two patterns of ROS related genes by cytoscape. **a** Upregulated; **b** Downregulated. **Fig. S9.** PPI network of two patterns of DERGs by cytoscape. **a** Upregulated; **b** Downregulated. **Fig. S10.** Power law distribution of node degree. **a** Degree distribution of ROS related PPI network; **b** Degree distribution of the DERGs PPI subnetwork. The graph displays a decreasing trend of degree distribution, with increasing number of links displaying scale-free topology. Black, red or blue curves represent total, upregulated, and downregulated PPI subnetworks, respectively. **Fig. S11.** RNA-seq analysis of 10 hub genes of DERGs of *H. undatus* peel with or without trypsin at 159 h of storage and RT-qPCR confirmation.

**Additional file 2: Fig. S12.** KEGG pathway of map 00940. Significant expressed genes were highlighted with blue borders.

**Additional file 3: Fig. S13.** KEGG pathway of map 04016. Significant expressed genes were highlighted with blue borders.

**Additional file 4: Table S1.** Number of transcripts or unigenes in 6 public databases.

**Additional file 5: Table S2.** Common unigenes in public databases including NR, Swiss-Prot, Pfam, COG, GO and KEGG.

**Additional file 6: Table S3.** Unigenes in functional groups based on COG and NOG classifications.

**Additional file 7: Table S4.** Number of unigenes enriched in KEGG pathways.

**Additional file 8: Table S5.** Information of unigenes shown in the volcano plot spectrum in Fig. 2a.

**Additional file 9: Table S6.** Common unigenes in different gene sets. All of the significantly expressed genes are named as allsignificant1703; 1117 ROS related genes are named as ROSall1117, including 434 upregulated and 465 downregulated genes; 85 DERGs are named as ROSsig85.

**Additional file 10: Table S7.** GO terms of DERGs by trypsin.

**Additional file 11: Table S8.** Full list of ROS related genes in each pattern after treatment with trypsin.

**Additional file 12: Table S9.** PPI network parameters of ROS related genes by Cytoscape.

**Additional file 13: Table S10.** PPI network parameters of upregulated ROS related genes by Cytoscape.

**Additional file 14: Table S11.** PPI network parameters of downregulated ROS related genes by Cytoscape.

**Additional file 15: Table S12.** Clusters of PPI network of ROS related genes by MCODE.

**Additional file 16: Table S13.** Top20 genes in DERGs network ranked by methods of MCC, degree, closeness or betweenness.

**Additional file 17: Table S14**. PPI network parameters of hub genes by Cytoscape.

**Additional file 18: Table S15.** Annotation of hub nodes of DERGs PPI network.

**Additional file 19: Table S16**. Information of hub genes.

## Data Availability

All data and materials used in this research are publicly available. Raw sequence data from this study have been submitted to the NCBI sequence read archive under the BioProject accession [PRJNA509494] and are available at the following link: https://trace.ncbi.nlm.nih.gov/Traces/sra_sub/sub.cgi?acc=SRP173572. Other supporting data are included as additional files listed below and are submitted with the manuscript.
