# Supplementary figures and images for: Transcriptomic analysis reveals hub genes and subnetworks related to ROS metabolism in Hylocereus undatus through novel superoxide scavenger trypsin treatment during storage
Source: BMC Genomics. 2020 Jun 26;21:437. doi: 10.1186/s12864-020-06850-1 (PMC7318492; doi:10.1186/s12864-020-06850-1)

**Supplementary figures**

Fig. S1


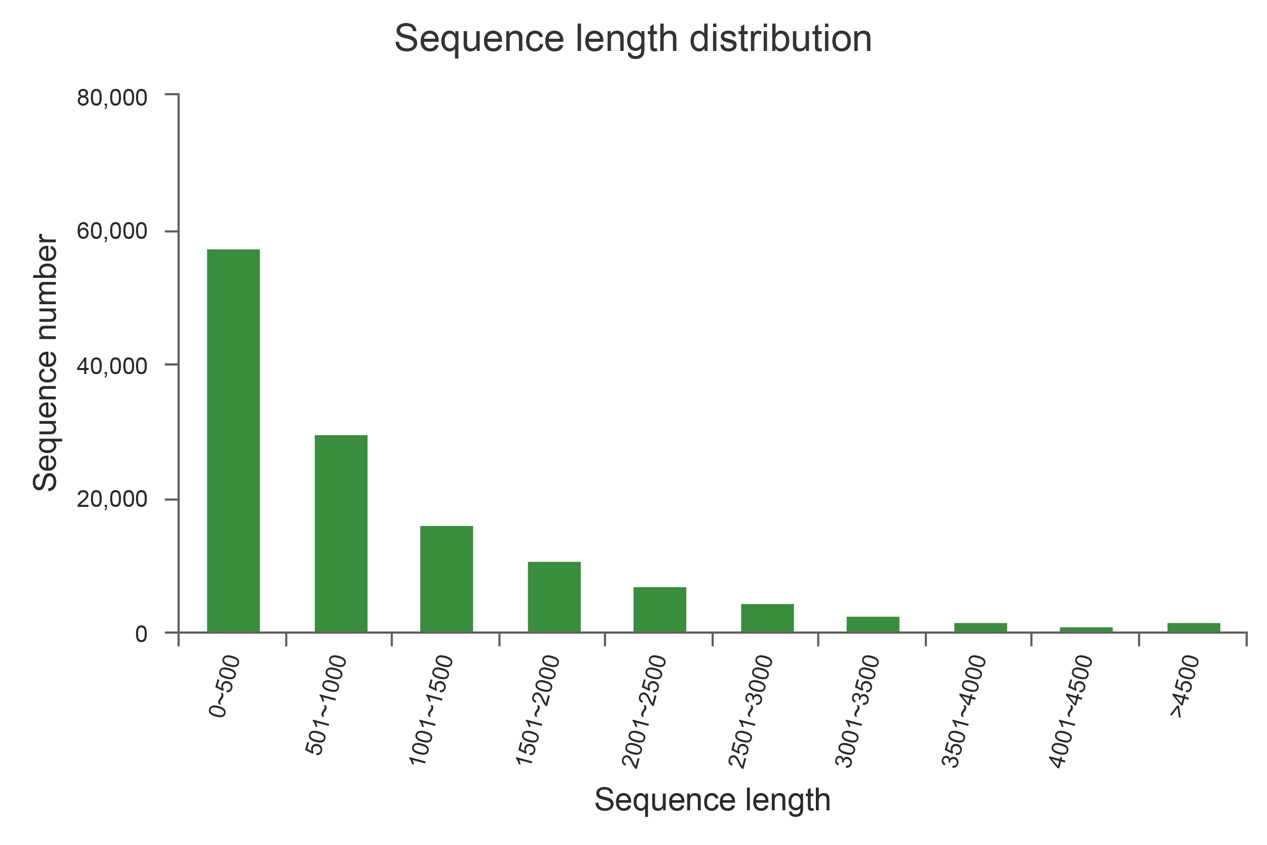


Fig. S2


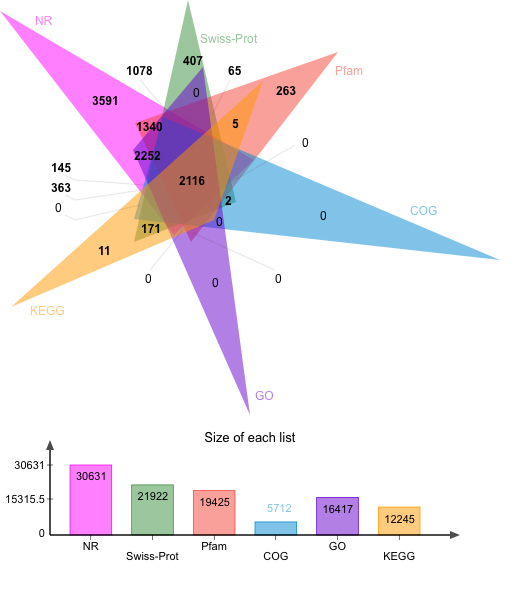


Fig. S3


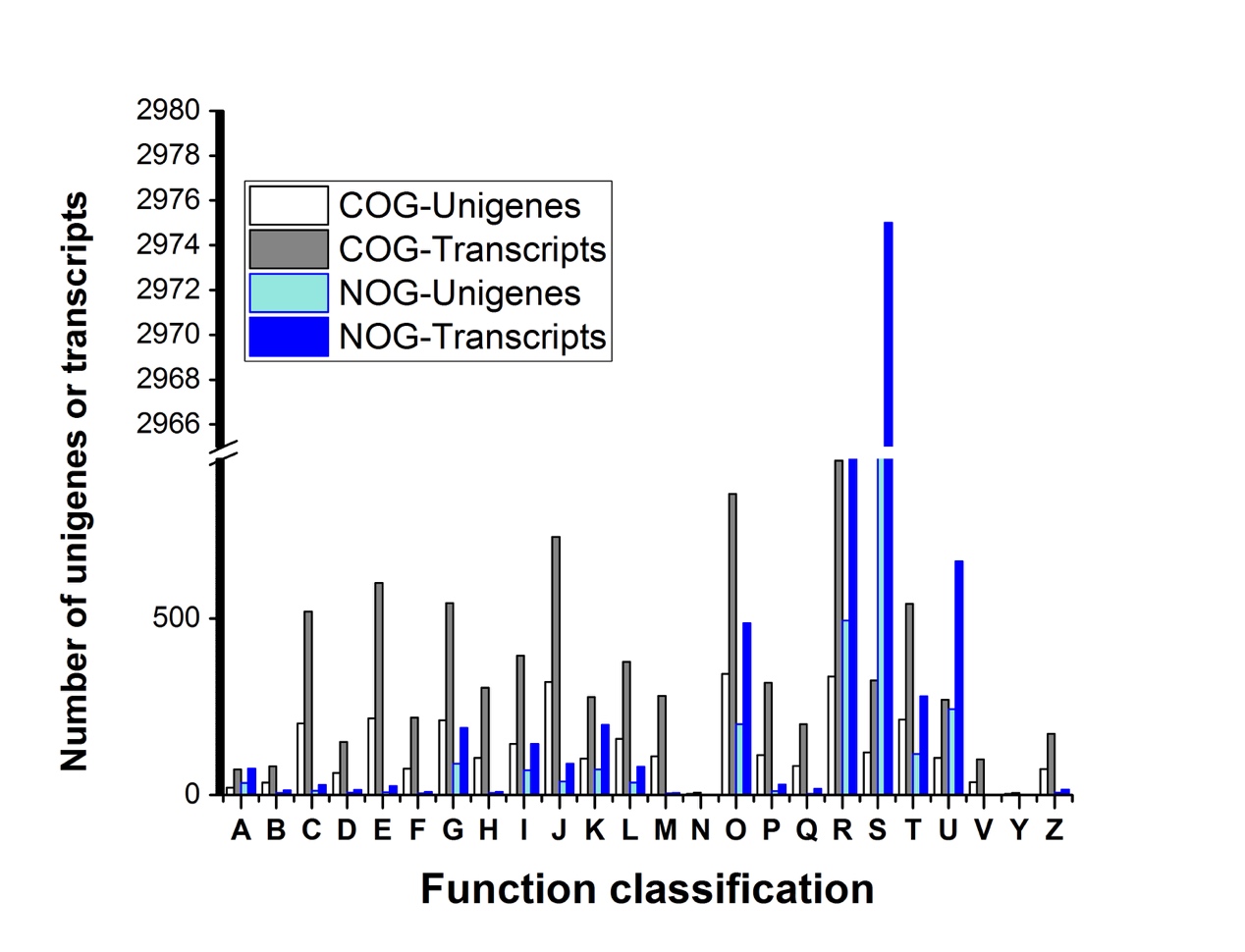


Fig. S4


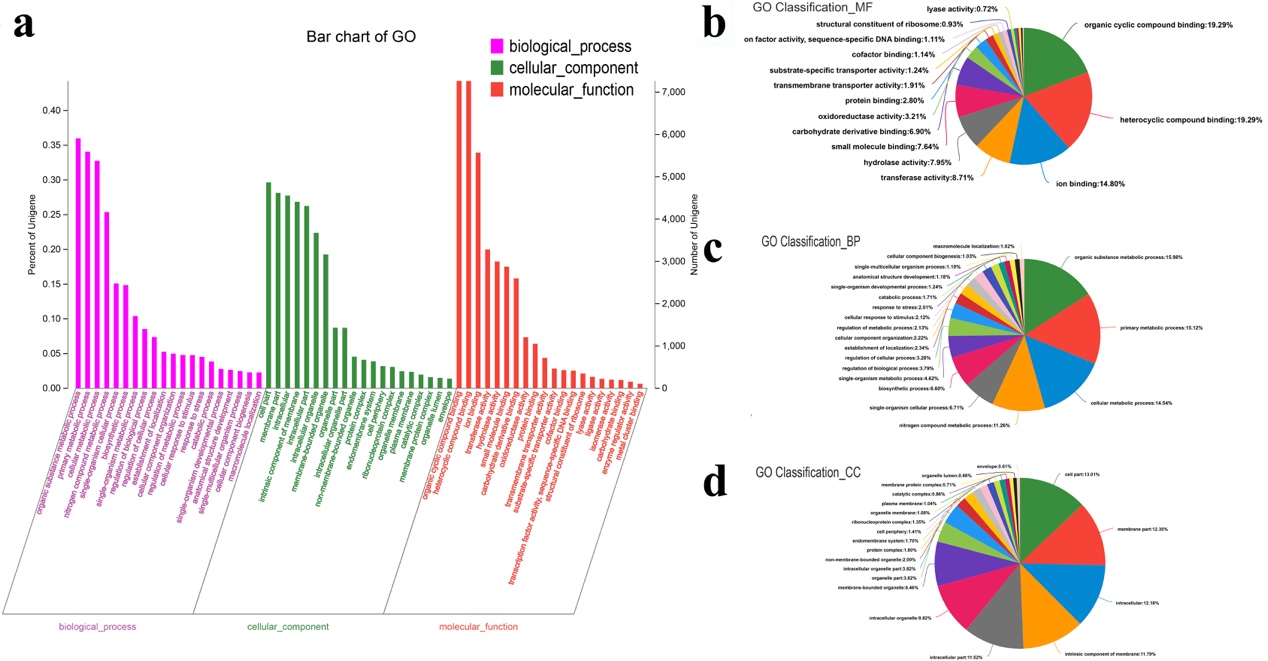


Fig. S5


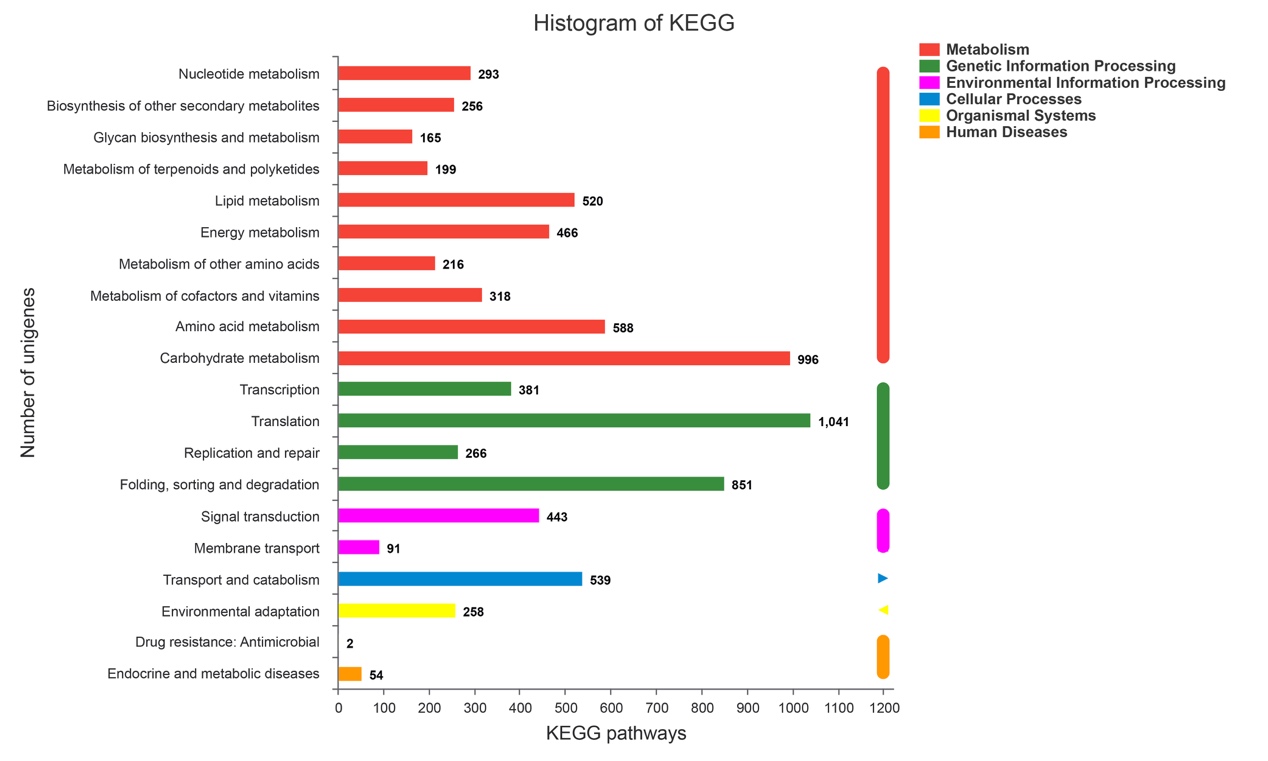


Fig. S6


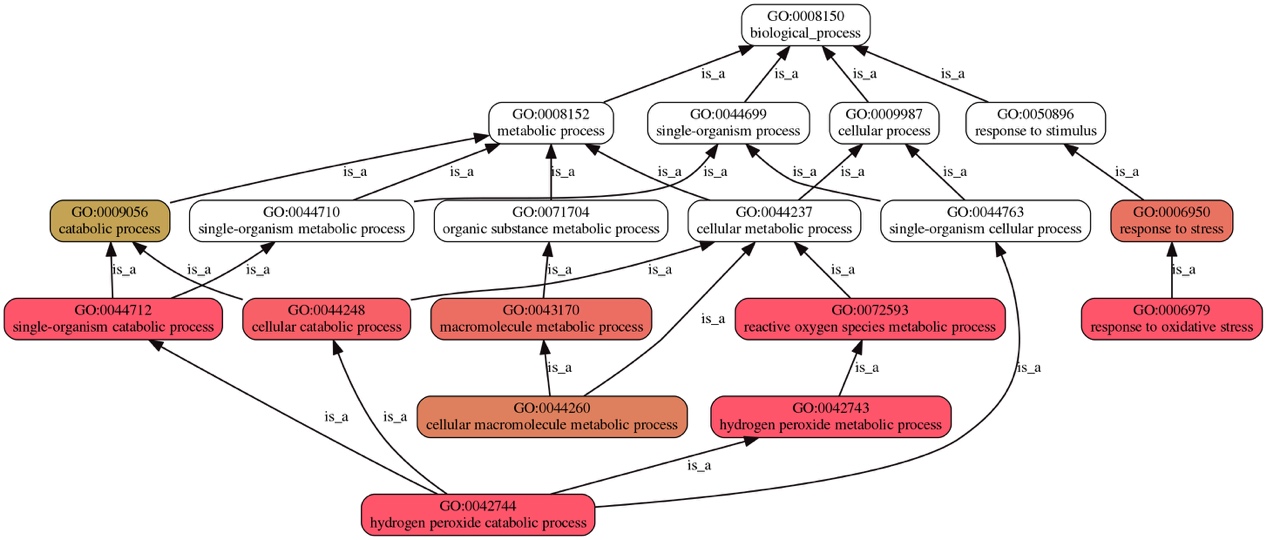


Fig. S7


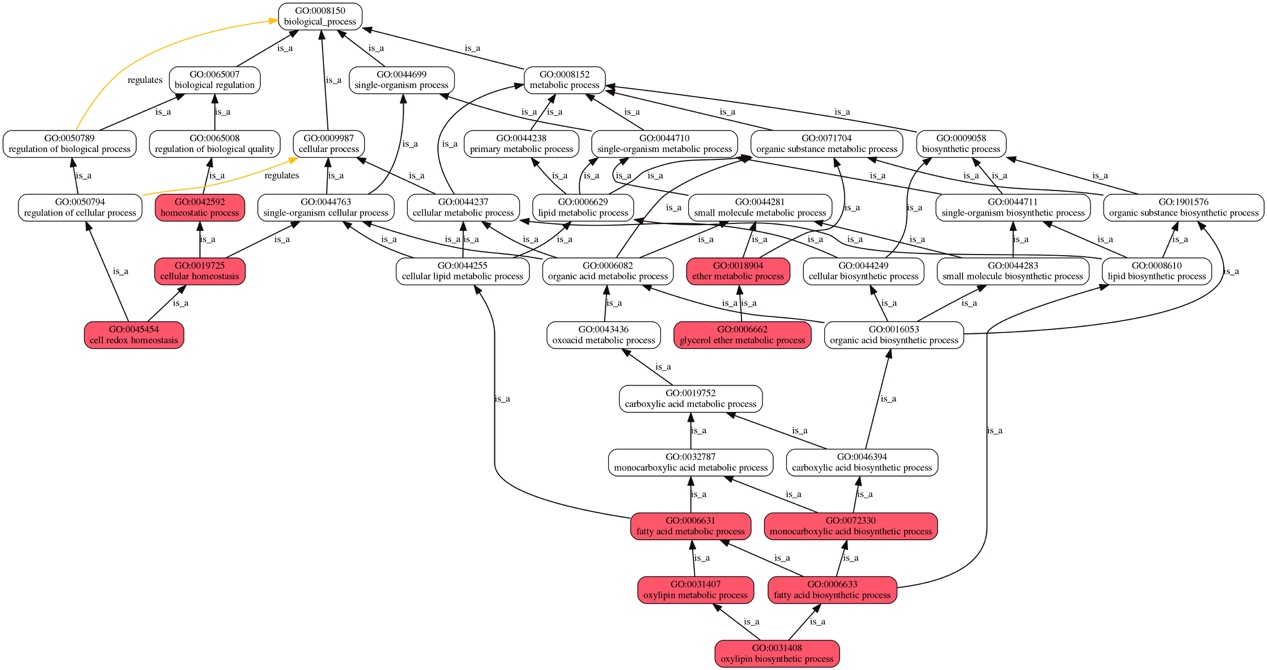


Fig. S8


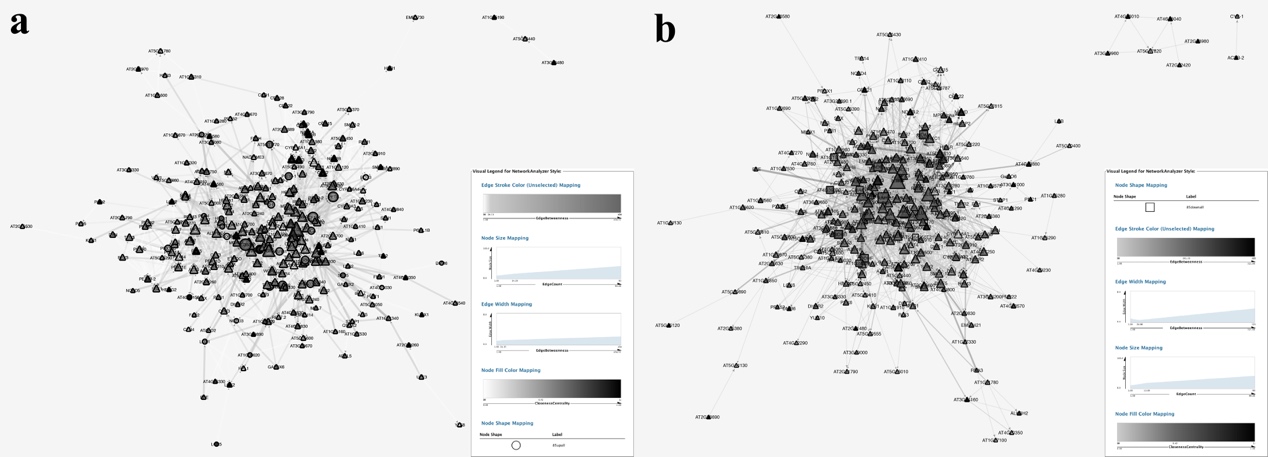


Fig. S9


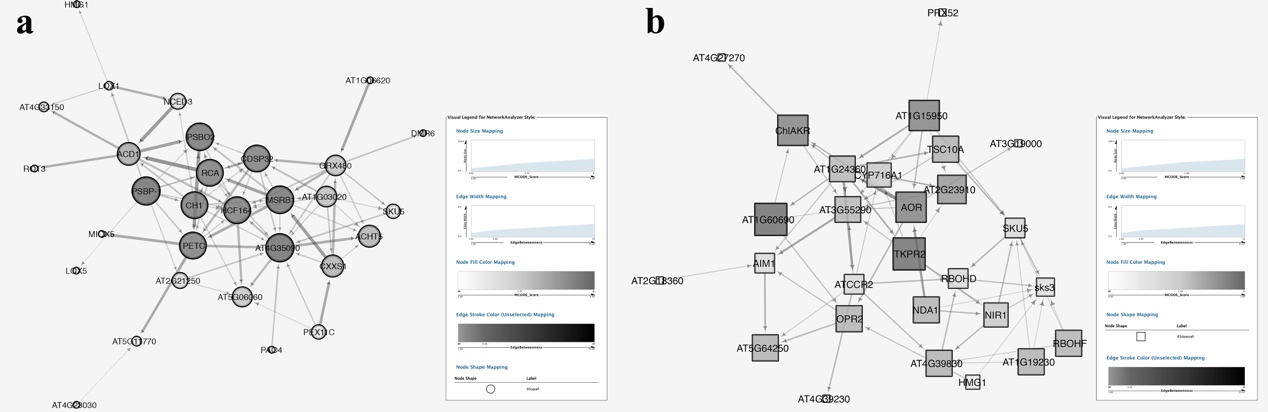


Fig. S10


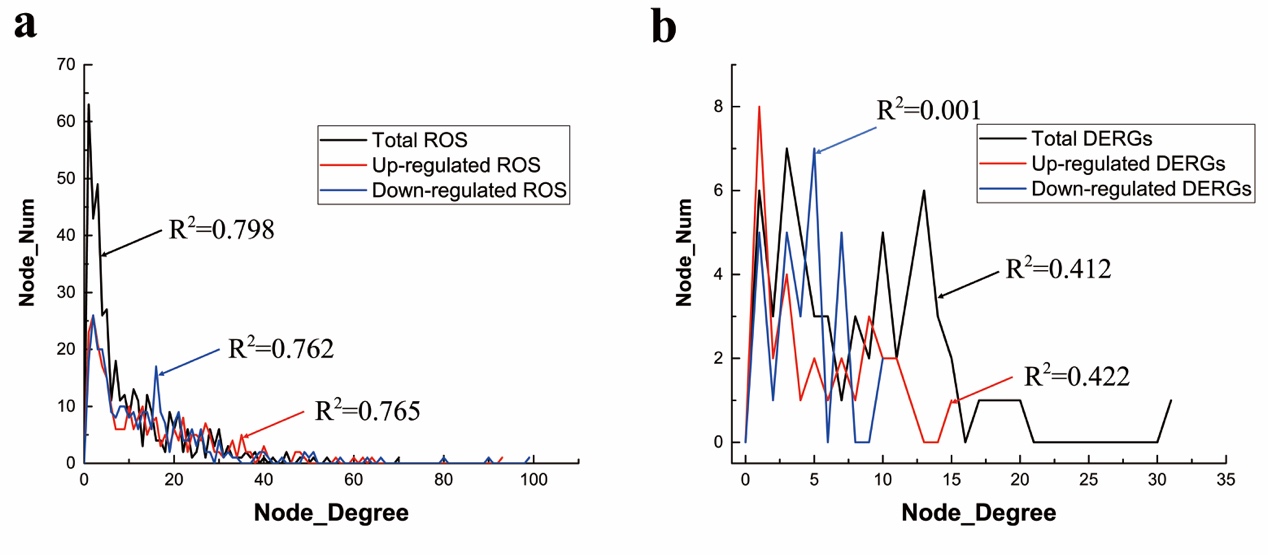


Fig. S11


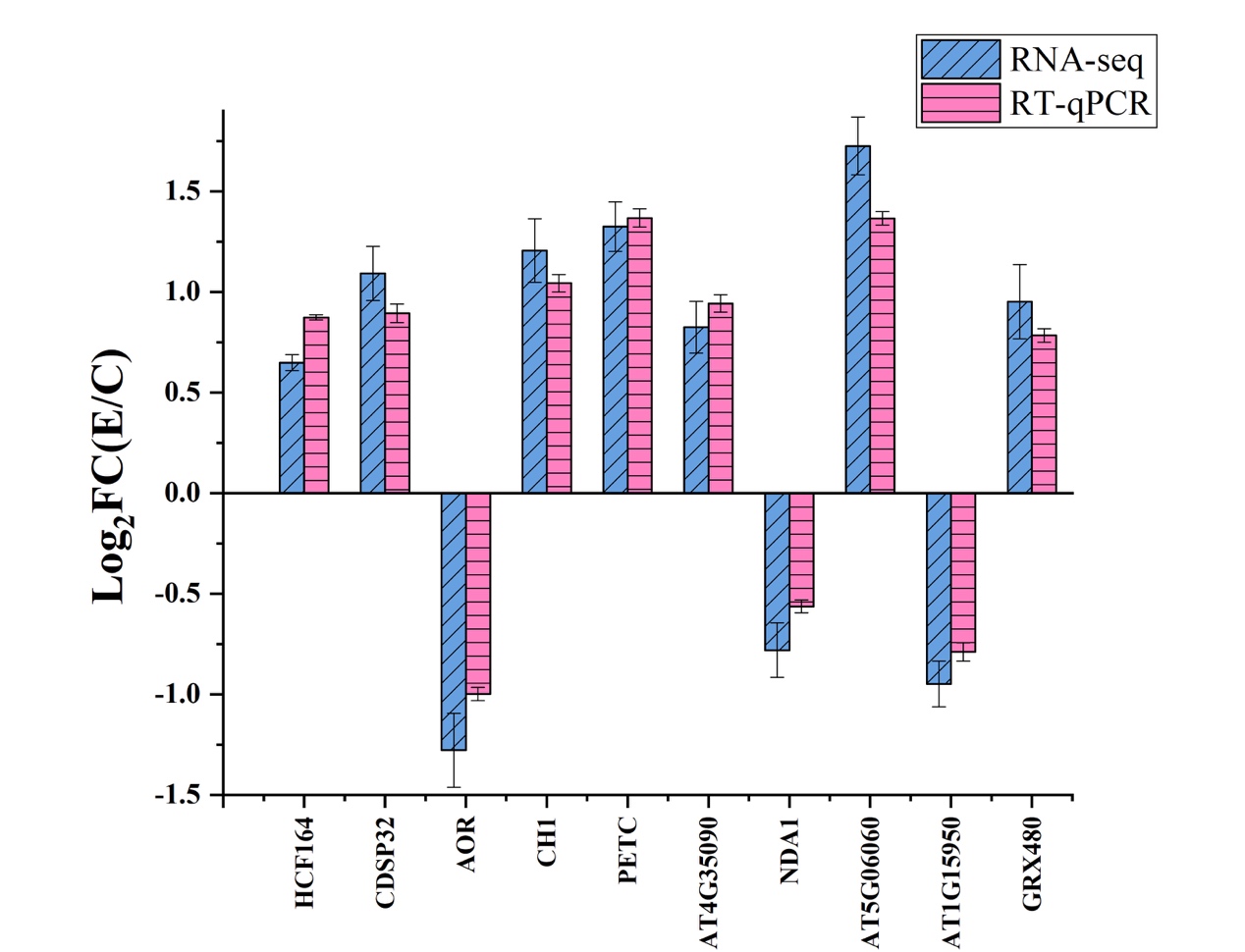

Supplement: Supplementary file 1 — Additional file 1: Fig. S1. Statistics of assembly length of transcripts. Fig. S2. Venn diagram of sequence statistics of functional annotation of RNA-Seq data for each database. Fig. S3. COG and NOG classification of transcripts or unigenes. Fig. S4. GO classification of unigenes. a Bar chart of three categories; (b-d) Molecular Function (MF), Biological Process (BP), or Cellular Component (CC) categories of unigenes. Fig. S5. Histogram of KEGG terms associated with trypsin. Different categories of KEGG terms were shown in different color. Red, Metabolism; Green, Genetic Information Processing; Purple, Environmental Information Processing; Blue, Cellular Processes; Yellow, HD, Organismal Systems; Brown, Human Diseases. Fig. S6. Relationships between GO terms of downregulated DERGs in a Directed Acyclic Graph (DAG). The information of color and arrow was same to that in Fig. 3. Fig. S7. Relationships between GO terms of upregulated DERGs in a Directed Acyclic Graph (DAG). The information of color and arrow was same to that in Fig. 3. Fig. S8. PPI network of two patterns of ROS related genes by cytoscape. a Upregulated; b Downregulated. Fig. S9. PPI network of two patterns of DERGs by cytoscape. a Upregulated; b Downregulated. Fig. S10. Power law distribution of node degree. a Degree distribution of ROS related PPI network; b Degree distribution of the DERGs PPI subnetwork. The graph displays a decreasing trend of degree distribution, with increasing number of links displaying scale-free topology. Black, red or blue curves represent total, upregulated, and downregulated PPI subnetworks, respectively. Fig. S11. RNA-seq analysis of 10 hub genes of DERGs of H. undatus peel with or without trypsin at 159 h of storage and RT-qPCR confirmation. [file 12864_2020_6850_MOESM1_ESM.docx]

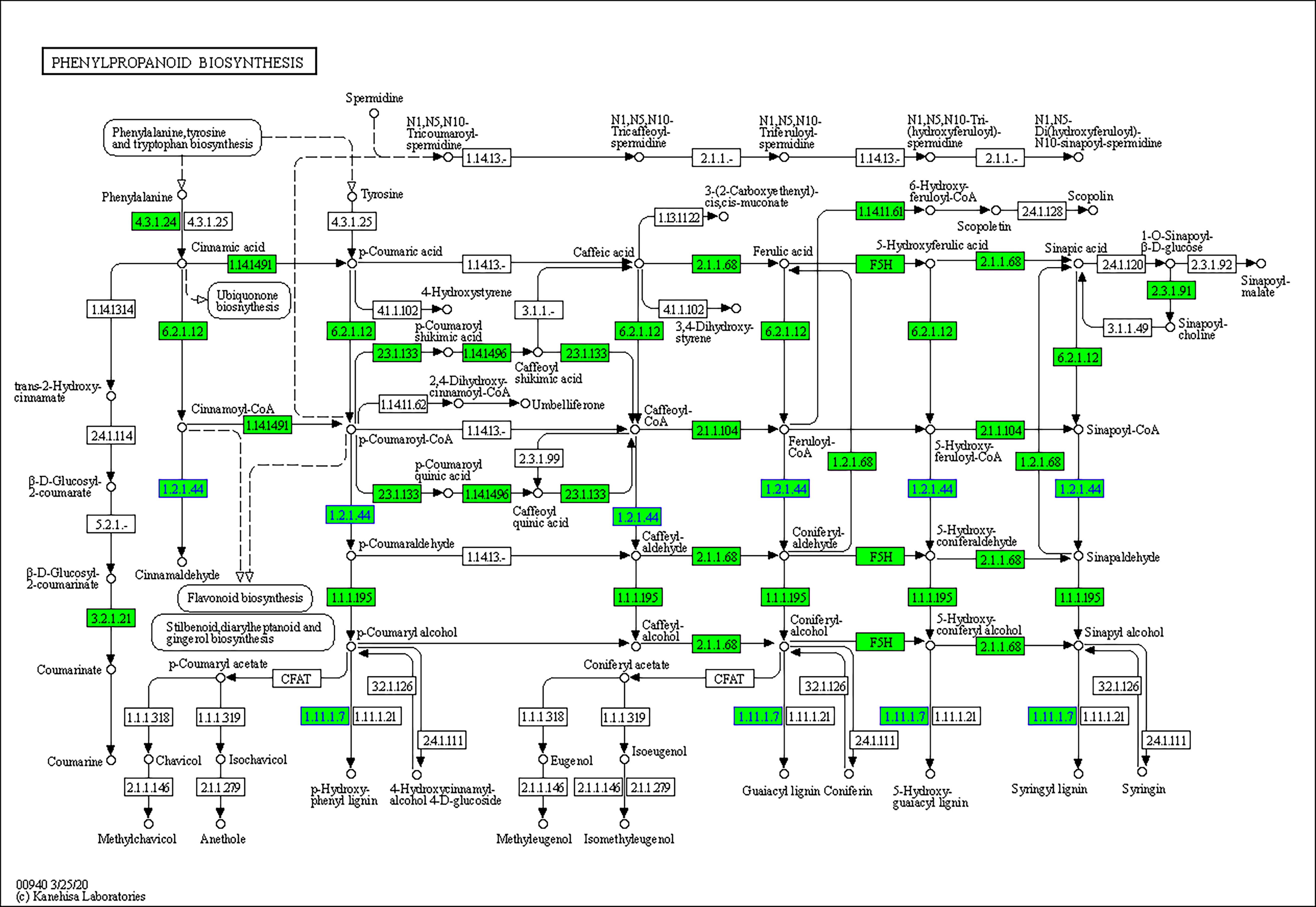

Supplement: Supplementary file 2 — Additional file 2: Fig. S12. KEGG pathway of map 00940. Significant expressed genes were highlighted with blue borders. [file 12864_2020_6850_MOESM2_ESM.jpg]

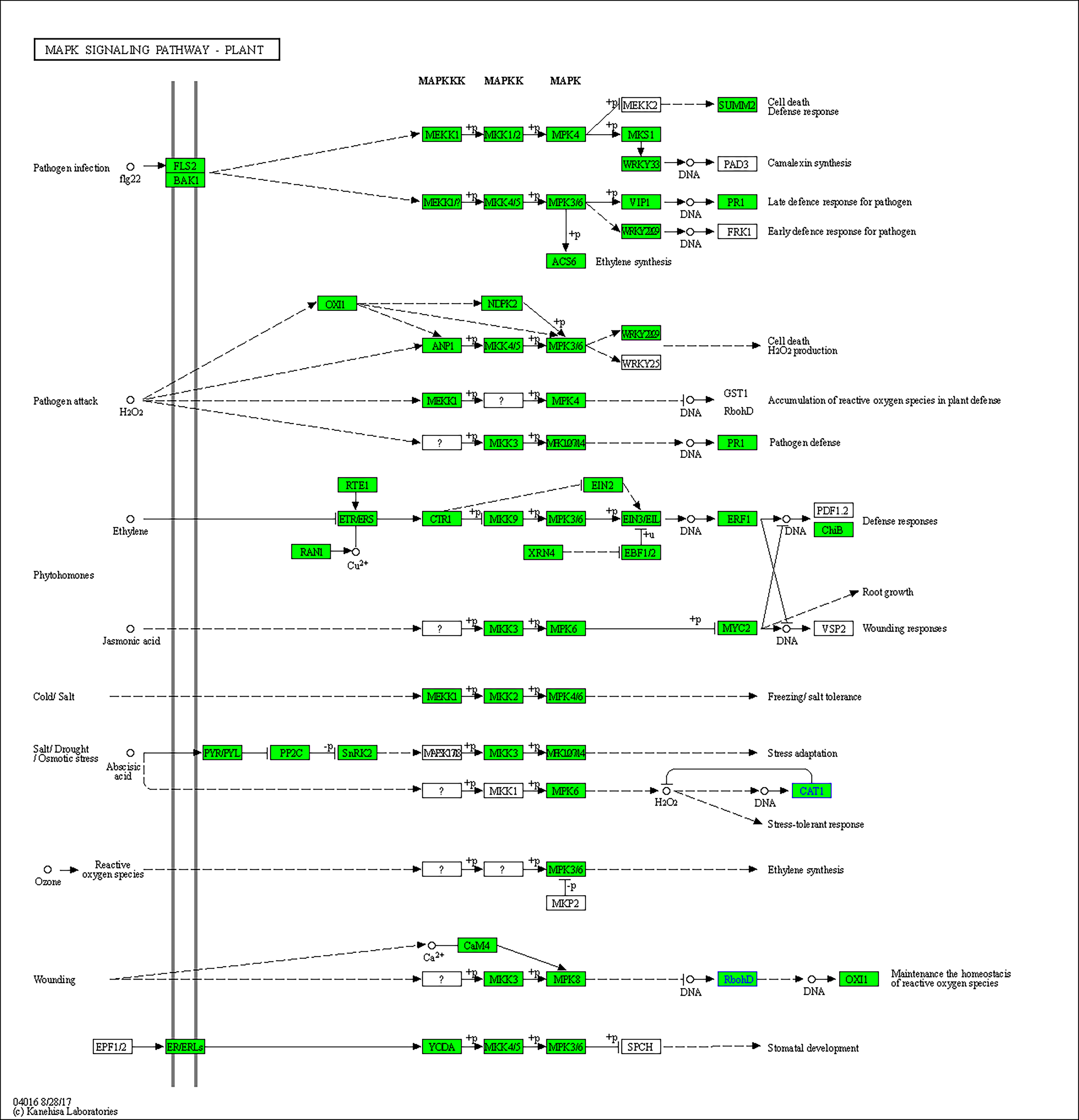

Supplement: Supplementary file 3 — Additional file 3: Fig. S13. KEGG pathway of map 04016. Significant expressed genes were highlighted with blue borders. [file 12864_2020_6850_MOESM3_ESM.jpg]
